# 2-Trifluoro­methyl-1*H*-benzimidazole

**DOI:** 10.1107/S1600536812017357

**Published:** 2012-04-25

**Authors:** Ming-Liang Liu

**Affiliations:** a Ordered Matter Science Research Center, College of Chemistry and Chemical Engineering, Southeast University, Nanjing 211189, People’s Republic of China

## Abstract

The asymmetric unit of the title compound, C_8_H_5_F_3_N_2_, consists of two half-mol­ecules, one lies on a mirror plane and the other is generated by twofold rotation symmetry, with the axis running through the trifluoro­methyl C atom and the attached benzimidazole C atom. The two 2-trifluoro­methyl-1*H*-benzimidazole mol­ecules are connected by N—H⋯N hydrogen bonds involving the disordered NH H atoms into chains running parallel to the *c* axis. One of the trifluoro­methyl groups is disordered over two orientations of equal occupancy.

## Related literature

For background to ferroelectric complexes, see: Fu *et al.* (2011 ▶); Zhang *et al.* (2010 ▶). For related structures, see: Liu (2011*a* ▶,*b* ▶, 2012*a* ▶,*b* ▶,*c* ▶). For graph-set analysis, see: Bernstein *et al.* (1995 ▶).
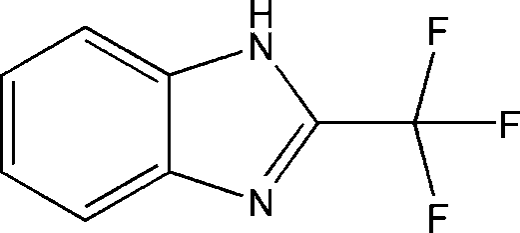


## Experimental

### 

#### Crystal data

C_8_H_5_F_3_N_2_
*M* *_r_* = 186.14
Orthorhombic, 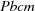

*a* = 11.859 (2) Å
*b* = 7.2154 (14) Å
*c* = 19.508 (4) Å
*V* = 1669.2 (5) Å^3^
*Z* = 8
Mo *K*α radiation
μ = 0.14 mm^−1^
*T* = 293 K
0.36 × 0.32 × 0.28 mm

#### Data collection

Rigaku SCXmini diffractometer
Absorption correction: multi-scan (*CrystalClear*; Rigaku, 2005 ▶) *T*_min_ = 0.952, *T*_max_ = 0.962
13301 measured reflections
1523 independent reflections
983 reflections with *I* > 2σ(*I*)
*R*_int_ = 0.074

#### Refinement

*R*[*F* ^2^ > 2σ(*F* ^2^)] = 0.071
*wR*(*F* ^2^) = 0.168
*S* = 1.04
1523 reflections
129 parameters
H-atom parameters constrained
Δρ_max_ = 0.28 e Å^−3^
Δρ_min_ = −0.23 e Å^−3^

### 

Data collection: *CrystalClear* (Rigaku, 2005 ▶); cell refinement: *CrystalClear*; data reduction: *CrystalClear*; program(s) used to solve structure: *SHELXS97* (Sheldrick, 2008 ▶); program(s) used to refine structure: *SHELXL97* (Sheldrick, 2008 ▶); molecular graphics: *PLATON* (Spek, 2009 ▶); software used to prepare material for publication: *SHELXL97*.

## Supplementary Material

Crystal structure: contains datablock(s) I, global. DOI: 10.1107/S1600536812017357/go2052sup1.cif

Structure factors: contains datablock(s) I. DOI: 10.1107/S1600536812017357/go2052Isup2.hkl

Supplementary material file. DOI: 10.1107/S1600536812017357/go2052Isup3.cml

Additional supplementary materials:  crystallographic information; 3D view; checkCIF report

## Figures and Tables

**Table 1:** Hydrogen-bond geometry (Å, °)

*D*—H⋯*A*	*D*—H	H⋯*A*	*D*⋯*A*	*D*—H⋯*A*
N1—H1*A*⋯N4	0.86	2.03	2.891 (3)	173
N4—H4*A*⋯N1	0.86	2.03	2.891 (3)	174

**Fig. 1.**
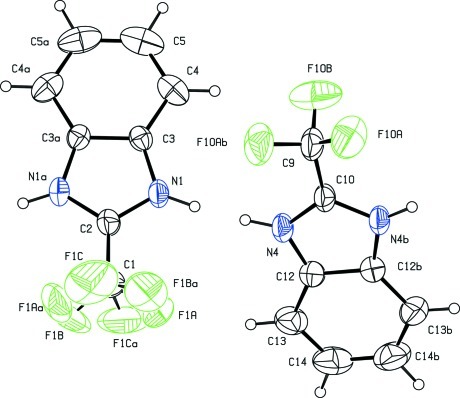
The molecular structure of the title compound, showing the atomic numbering scheme with 30% probability displacement ellipsoids.Both half hydrogens attached to N1 and N2 and the disordered fluorine atoms are included.

**Fig. 2.**
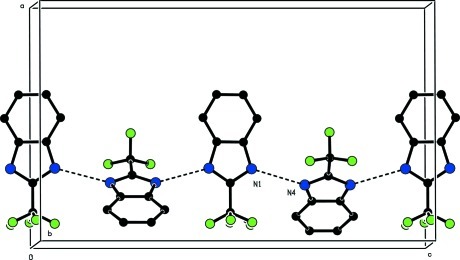
View of the C3 chain running parallel to the c-axis. For clarity all hydrogen atoms are omitted.

## supplementary crystallographic information

### Comment

Recently much attention has been devoted to crystals containing organic ions and inorganic ions due to the possibility of tuning their special structural features and their potential ferroelectrics properties (Fu *et al.*, 2011; Zhang *et al.*, 2010.). In our laboratory, the title compound has been synthesized to investigate to its potential ferroelectric properties. However, it was found that the dielectric constant of the compound as a function of temperature indicates that the permittivity is basically temperature-independent (*ε* = C/(T–T_0_)), suggesting that this compound is not ferroelectric or there may be no distinct phase transition occurring within the measured temperature (below the melting point).

The title compound has an asymmetric unit that consists of two half 2-trifluoromethyl-1*H*-benzimidazole molecules (Fig 1). In each of these molecules the H atoms attached to the N atoms are shared 50/50 over both sites.

One of these molecule sits on a mirror plane at *c* = 0.75 and the other sits on a 2-fold axis at *b* = 0.25 and *c* = 0.5 with the axis running through atoms C1 and C2 of the trifluoromethyl group.

The molecules of, I, are hydrogen bonded together to form C^2^_3_(8) chain,(Bernstein *et al.*, 1995), which run parallel to the c-axis. Half by N1···N4, N4···N1 and N1..N4 chains. and half by N4···N1, N1···N4 and N4···N1 chaims, (in each case the first atom is the donor and the second the acceptor).

One of the trifluoromethyl groups is disordered.

### Experimental

0.144 g (1 mmol) of 2-trifluoromethyl-1*H*-benzimidazole was dissolved in 30 ml of ethanol to give a solution at the ambient temperature. Single crystals suitable for X-ray structure analysis were obtained by the slow evaporation of the above solution after 3 days in air.

### Refinement

H atoms were treated as riding atoms with N—H, 0.86Å, C—H(aromatic), 0.95 Å, with *U*_iso_ = 1.2Ueq(C) allowed to ride. An examination of a difference map along the line of the N1 to N2 vector showed an elongated density peak. This was found to be best modelled as two half-hydrogen atoms attached to N1 and N4. These positions were checked on the final difference map.

The disordered trifluoromethyl was modelled with restrained bonds and angles based on the average values found for the non-disordered trifluoromethyl group with initial positions being derived from a difference map. The action of the symmetry axis passing molecule produced a set of six F atoms spaced around a regular hexagon. Each of these F atoms was given a site occupancy of 0.5. In the final stages of refinement the group was refined as a riding and rotating group as for a methyl group. This model is not perfect and as a result there are several C Alerts.

### Crystal data

**Table:**

C_8_H_5_F_3_N_2_	*F*(000) = 752
*M**_r_* = 186.14	*D*_x_ = 1.481 Mg m^−^^3^
Orthorhombic, *P**b**c**m*	Mo *K*α radiation, λ = 0.71073 Å
Hall symbol: -P 2c 2b	θ = 0–25°
*a* = 11.859 (2) Å	µ = 0.14 mm^−^^1^
*b* = 7.2154 (14) Å	*T* = 293 K
*c* = 19.508 (4) Å	Block, colourless
*V* = 1669.2 (5) Å^3^	0.36 × 0.32 × 0.28 mm
*Z* = 8	

### Data collection

**Table:**

Rigaku SCXmini diffractometer	1523 independent reflections
Radiation source: fine-focus sealed tube	983 reflections with *I* > 2σ(*I*)
Graphite monochromator	*R*_int_ = 0.074
CCD_Profile_fitting scans	θ_max_ = 25.0°, θ_min_ = 3.3°
Absorption correction: multi-scan (*CrystalClear*; Rigaku, 2005)	*h* = −14→14
*T*_min_ = 0.952, *T*_max_ = 0.962	*k* = −8→8
13301 measured reflections	*l* = −23→22

### Refinement

**Table:**

Refinement on *F*^2^	Primary atom site location: structure-invariant direct methods
Least-squares matrix: full	Secondary atom site location: difference Fourier map
*R*[*F*^2^ > 2σ(*F*^2^)] = 0.071	Hydrogen site location: inferred from neighbouring sites
*wR*(*F*^2^) = 0.168	H-atom parameters constrained
*S* = 1.04	*w* = 1/[σ^2^(*F*_o_^2^) + (0.0577*P*)^2^ + 1.2173*P*] where *P* = (*F*_o_^2^ + 2*F*_c_^2^)/3
1523 reflections	(Δ/σ)_max_ < 0.001
129 parameters	Δρ_max_ = 0.28 e Å^−^^3^
0 restraints	Δρ_min_ = −0.23 e Å^−^^3^

### Special details

**Table:**

Geometry. All esds (except the esd in the dihedral angle between two l.s. planes) are estimated using the full covariance matrix. The cell esds are taken into account individually in the estimation of esds in distances, angles and torsion angles; correlations between esds in cell parameters are only used when they are defined by crystal symmetry. An approximate (isotropic) treatment of cell esds is used for estimating esds involving l.s. planes.
Refinement. Refinement of F^2^ against ALL reflections. The weighted R-factor wR and goodness of fit S are based on F^2^, conventional R-factors R are based on F, with F set to zero for negative F^2^. The threshold expression of F^2^ > 2sigma(F^2^) is used only for calculating R-factors(gt) etc. and is not relevant to the choice of reflections for refinement. R-factors based on F^2^ are statistically about twice as large as those based on F, and R- factors based on ALL data will be even larger.

### Fractional atomic coordinates and isotropic or equivalent isotropic displacement parameters (Å^2^)

**Table:**

	*x*	*y*	*z*	*U*_iso_*/*U*_eq_	Occ. (<1)
C2	0.2645 (4)	0.2500	0.5000	0.0595 (12)	
C1	0.14185 (18)	0.2500	0.5000	0.101 (2)	
F1A	0.09271 (18)	0.1770	0.5534	0.157 (4)	0.50
F1B	0.09835 (18)	0.1655	0.4469	0.191 (5)	0.50
F1C	0.10542 (18)	0.4210	0.4964	0.213 (5)	0.50
N1	0.3246 (2)	0.2091 (3)	0.55507 (11)	0.0576 (7)	
H1A	0.3000	0.1795	0.5951	0.069*	0.50
C3	0.4353 (2)	0.2242 (4)	0.53443 (14)	0.0495 (7)	
C4	0.5347 (3)	0.1964 (5)	0.5697 (2)	0.0760 (11)	
H4	0.5350	0.1606	0.6155	0.091*	
C5	0.6321 (3)	0.2236 (6)	0.5345 (2)	0.0986 (16)	
H5	0.7006	0.2067	0.5568	0.118*	
F10A	0.3495 (3)	0.4573 (4)	0.80332 (12)	0.1403 (12)	
F10B	0.4734 (4)	0.3146 (6)	0.7500	0.1557 (19)	
N4	0.2610 (2)	0.1033 (4)	0.69268 (12)	0.0641 (8)	
H4A	0.2749	0.1364	0.6512	0.077*	0.50
C9	0.3662 (6)	0.3519 (8)	0.7500	0.0806 (17)	
C10	0.2953 (4)	0.1861 (6)	0.7500	0.0605 (12)	
C12	0.1984 (3)	−0.0467 (5)	0.71420 (15)	0.0623 (9)	
C13	0.1394 (3)	−0.1800 (6)	0.6772 (2)	0.0855 (12)	
H13	0.1382	−0.1800	0.6295	0.103*	
C14	0.0830 (4)	−0.3113 (7)	0.7148 (2)	0.1073 (16)	
H14	0.0434	−0.4036	0.6917	0.129*	

### Atomic displacement parameters (Å^2^)

**Table:**

	*U*^11^	*U*^22^	*U*^33^	*U*^12^	*U*^13^	*U*^23^
C2	0.059 (3)	0.076 (3)	0.044 (3)	0.000	0.000	−0.002 (2)
C1	0.066 (4)	0.134 (6)	0.102 (5)	0.000	0.000	0.004 (4)
F1A	0.078 (4)	0.304 (13)	0.088 (5)	−0.044 (9)	0.024 (5)	0.041 (5)
F1B	0.075 (5)	0.376 (17)	0.121 (6)	−0.035 (10)	−0.039 (5)	−0.054 (7)
F1C	0.089 (5)	0.263 (10)	0.288 (12)	0.077 (6)	0.034 (12)	0.078 (11)
N1	0.0703 (17)	0.0672 (18)	0.0354 (13)	−0.0017 (13)	−0.0008 (13)	0.0038 (11)
C3	0.0575 (18)	0.0449 (17)	0.0461 (15)	0.0004 (14)	−0.0057 (14)	−0.0042 (13)
C4	0.082 (3)	0.062 (2)	0.084 (2)	0.0088 (19)	−0.026 (2)	−0.0089 (19)
C5	0.070 (2)	0.068 (3)	0.158 (5)	0.011 (2)	−0.030 (2)	−0.033 (3)
F10A	0.216 (3)	0.113 (2)	0.0923 (18)	−0.069 (2)	0.0224 (18)	−0.0384 (15)
F10B	0.097 (3)	0.117 (3)	0.252 (6)	−0.040 (3)	0.000	0.000
N4	0.081 (2)	0.0755 (19)	0.0357 (13)	−0.0105 (15)	0.0007 (13)	−0.0028 (13)
C9	0.117 (5)	0.082 (4)	0.043 (3)	−0.027 (4)	0.000	0.000
C10	0.076 (3)	0.069 (3)	0.037 (2)	−0.010 (3)	0.000	0.000
C12	0.059 (2)	0.073 (2)	0.0549 (17)	−0.0047 (17)	0.0013 (15)	−0.0026 (16)
C13	0.076 (3)	0.098 (3)	0.083 (3)	−0.012 (2)	−0.001 (2)	−0.025 (2)
C14	0.087 (3)	0.099 (3)	0.135 (4)	−0.030 (2)	−0.005 (2)	−0.022 (3)

### Geometric parameters (Å, º)

**Table:**

C2—N1^i^	1.323 (3)	C5—H5	0.9300
C2—N1	1.323 (3)	F10A—C9	1.303 (4)
C2—C1	1.454 (5)	F10B—C9	1.300 (7)
C1—F1A^i^	1.3052	N4—C10	1.331 (3)
C1—F1A	1.3053	N4—C12	1.378 (4)
C1—F1B	1.3079	N4—H4A	0.8600
C1—F1B^i^	1.3079	C9—F10A^ii^	1.304 (4)
C1—F1C^i^	1.3095	C9—C10	1.462 (7)
C1—F1C	1.3095	C10—N4^ii^	1.331 (3)
N1—C3	1.377 (4)	C12—C13	1.392 (5)
N1—H1A	0.8600	C12—C12^ii^	1.397 (6)
C3—C4	1.380 (4)	C13—C14	1.372 (6)
C3—C3^i^	1.394 (5)	C13—H13	0.9300
C4—C5	1.359 (5)	C14—C14^ii^	1.375 (9)
C4—H4	0.9300	C14—H14	0.9300
C5—C5^i^	1.399 (9)		
			
N1^i^—C2—N1	114.7 (4)	C4—C5—C5^i^	121.8 (2)
N1^i^—C2—C1	122.6 (2)	C4—C5—H5	119.1
N1—C2—C1	122.6 (2)	C5^i^—C5—H5	119.1
F1A—C1—F1B	105.6	C10—N4—C12	105.1 (3)
F1A^i^—C1—F1B^i^	105.6	C10—N4—H4A	127.4
F1A^i^—C1—F1C^i^	106.0	C12—N4—H4A	127.4
F1B^i^—C1—F1C^i^	105.4	F10B—C9—F10A	105.6 (4)
F1A—C1—F1C	106.0	F10B—C9—F10A^ii^	105.6 (4)
F1B—C1—F1C	105.4	F10A—C9—F10A^ii^	105.9 (5)
F1A^i^—C1—C2	116.522 (6)	F10B—C9—C10	113.1 (5)
F1A—C1—C2	116.519 (5)	F10A—C9—C10	113.0 (3)
F1B—C1—C2	113.229 (5)	F10A^ii^—C9—C10	113.0 (3)
F1B^i^—C1—C2	113.230 (5)	N4—C10—N4^ii^	114.3 (4)
F1C^i^—C1—C2	109.263 (5)	N4—C10—C9	122.9 (2)
F1C—C1—C2	109.262 (5)	N4^ii^—C10—C9	122.9 (2)
C2—N1—C3	105.0 (3)	N4—C12—C13	131.0 (3)
C2—N1—H1A	127.5	N4—C12—C12^ii^	107.74 (16)
C3—N1—H1A	127.5	C13—C12—C12^ii^	121.3 (2)
N1—C3—C4	131.0 (3)	C14—C13—C12	116.4 (4)
N1—C3—C3^i^	107.64 (15)	C14—C13—H13	121.8
C4—C3—C3^i^	121.3 (2)	C12—C13—H13	121.8
C5—C4—C3	116.9 (4)	C13—C14—C14^ii^	122.3 (2)
C5—C4—H4	121.5	C13—C14—H14	118.9
C3—C4—H4	121.5	C14^ii^—C14—H14	118.9
			
N1^i^—C2—C1—F1A^i^	−11.43 (13)	N1—C3—C4—C5	−179.7 (3)
N1—C2—C1—F1A^i^	168.57 (13)	C3^i^—C3—C4—C5	0.7 (5)
N1^i^—C2—C1—F1A	168.57 (13)	C3—C4—C5—C5^i^	−0.4 (7)
N1—C2—C1—F1A	−11.43 (13)	C12—N4—C10—N4^ii^	0.2 (5)
N1^i^—C2—C1—F1B	45.84 (13)	C12—N4—C10—C9	179.2 (5)
N1—C2—C1—F1B	−134.16 (13)	F10B—C9—C10—N4	−89.5 (4)
N1^i^—C2—C1—F1B^i^	−134.16 (13)	F10A—C9—C10—N4	150.6 (4)
N1—C2—C1—F1B^i^	45.84 (13)	F10A^ii^—C9—C10—N4	30.4 (8)
N1^i^—C2—C1—F1C^i^	108.66 (13)	F10B—C9—C10—N4^ii^	89.5 (4)
N1—C2—C1—F1C^i^	−71.34 (13)	F10A—C9—C10—N4^ii^	−30.4 (8)
N1^i^—C2—C1—F1C	−71.34 (13)	F10A^ii^—C9—C10—N4^ii^	−150.6 (4)
N1—C2—C1—F1C	108.66 (13)	C10—N4—C12—C13	178.1 (4)
N1^i^—C2—N1—C3	−0.07 (14)	C10—N4—C12—C12^ii^	−0.1 (3)
C1—C2—N1—C3	179.93 (14)	N4—C12—C13—C14	−178.8 (4)
C2—N1—C3—C4	−179.4 (3)	C12^ii^—C12—C13—C14	−0.8 (4)
C2—N1—C3—C3^i^	0.2 (3)	C12—C13—C14—C14^ii^	0.8 (4)

Symmetry codes: (i) *x*, −*y*+1/2, −*z*+1; (ii) *x*, *y*, −*z*+3/2.

### Hydrogen-bond geometry (Å, º)

**Table:**

*D*—H···*A*	*D*—H	H···*A*	*D*···*A*	*D*—H···*A*
N1—H1*A*···N4	0.86	2.03	2.891 (3)	173
N4—H4*A*···N1	0.86	2.03	2.891 (3)	174
